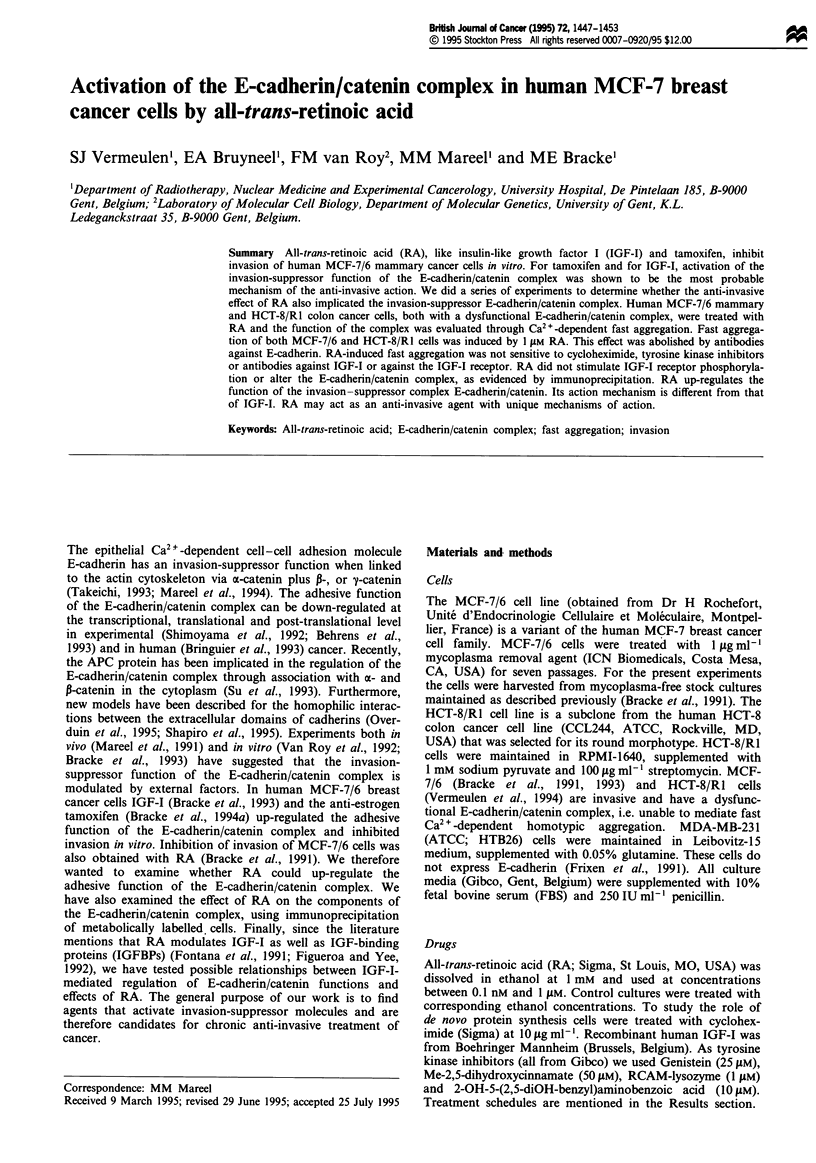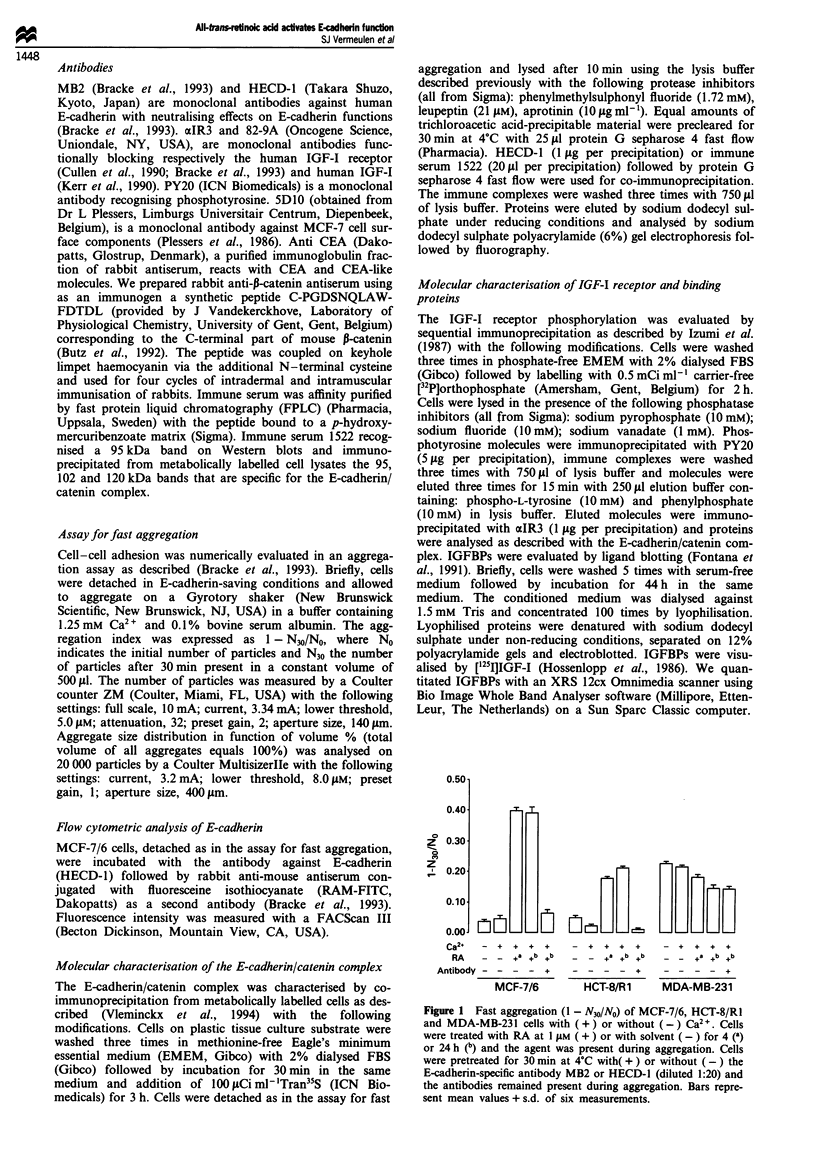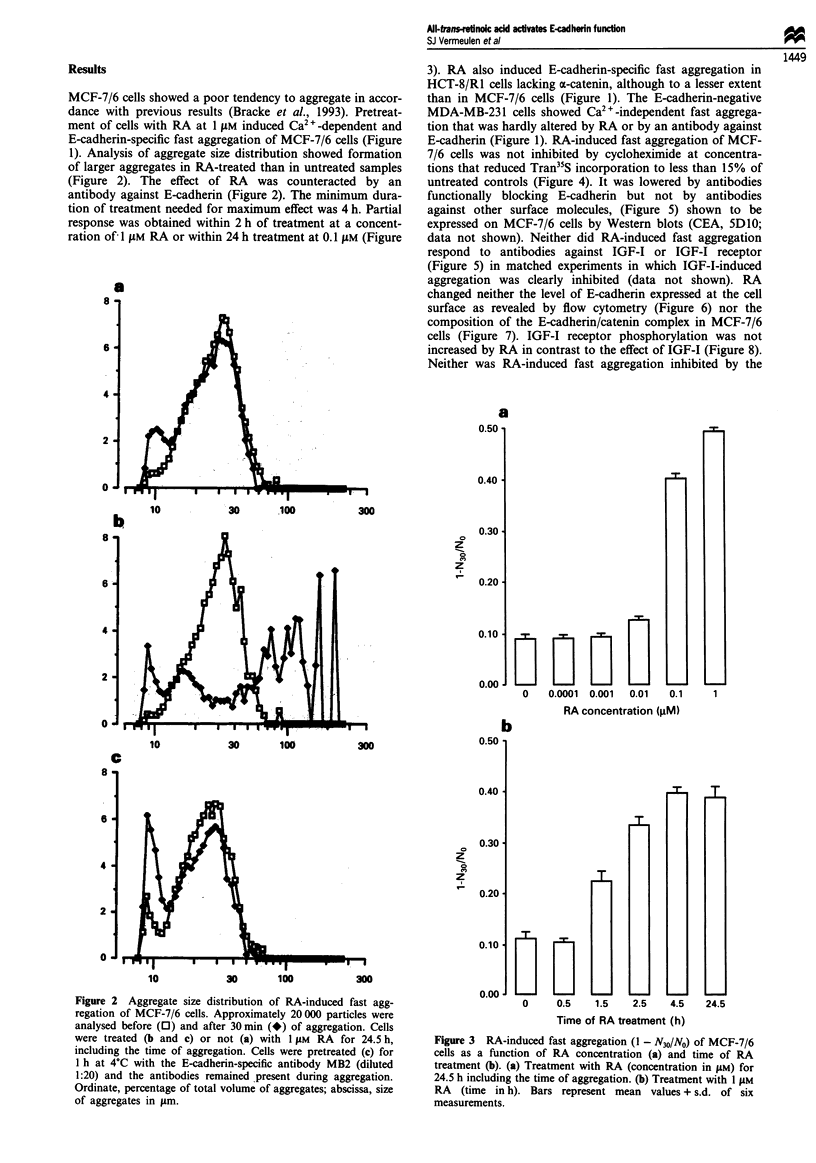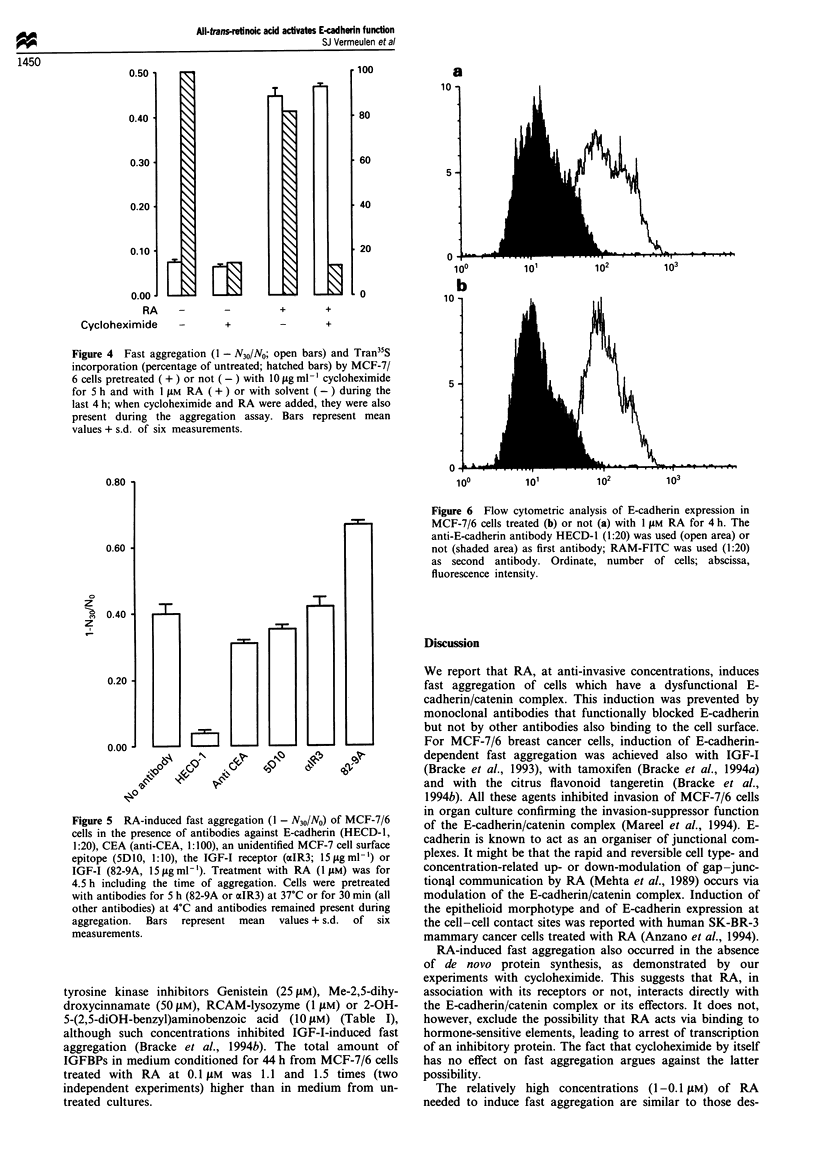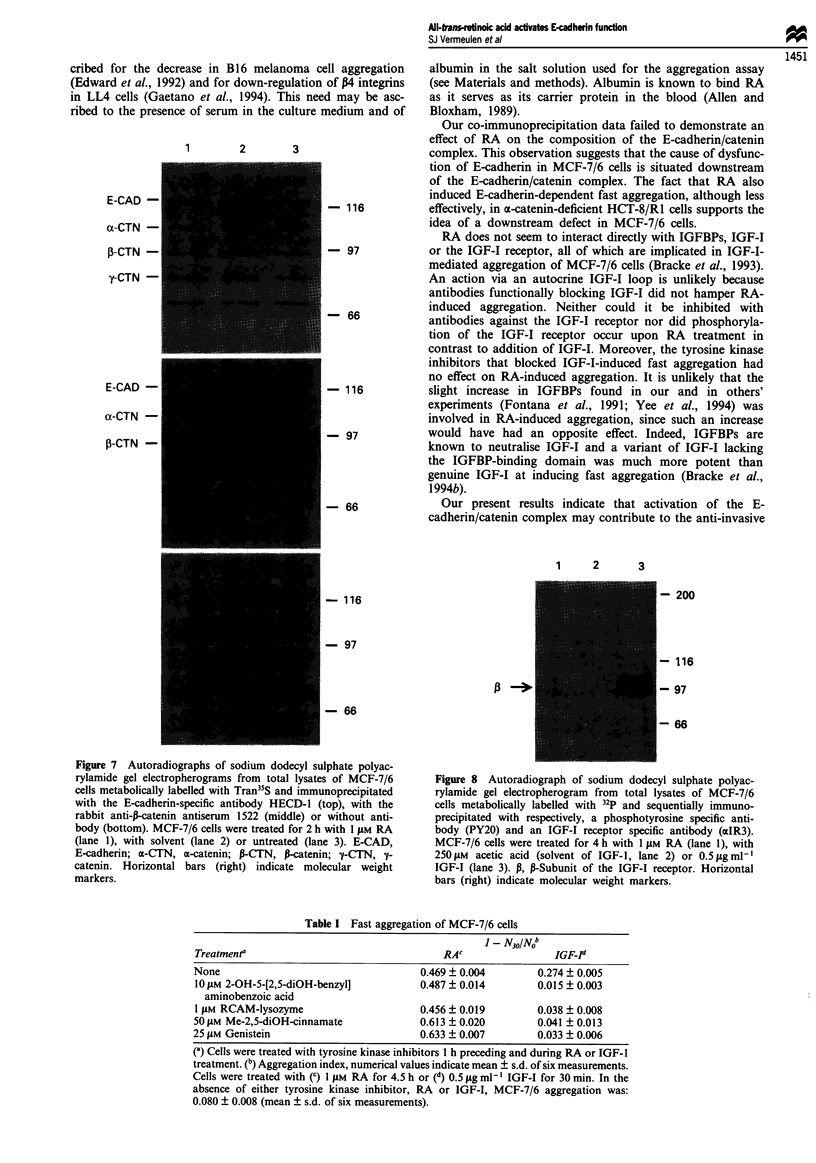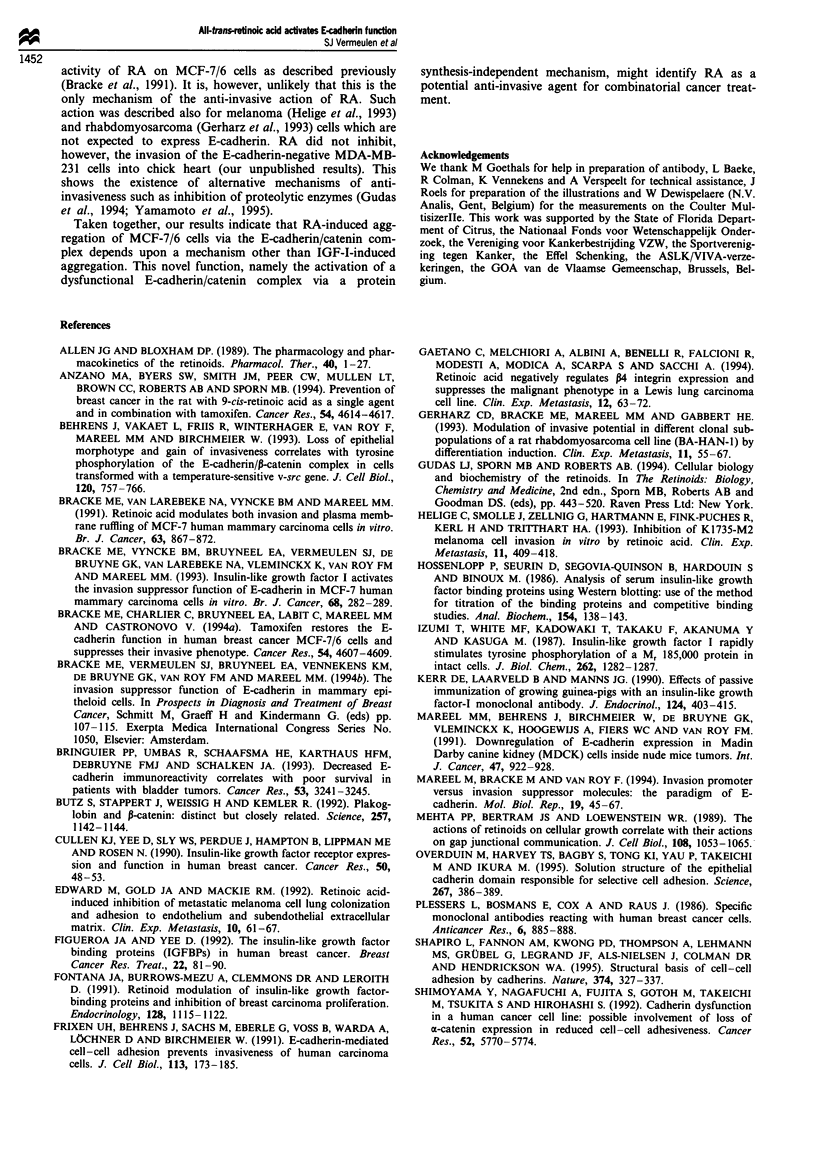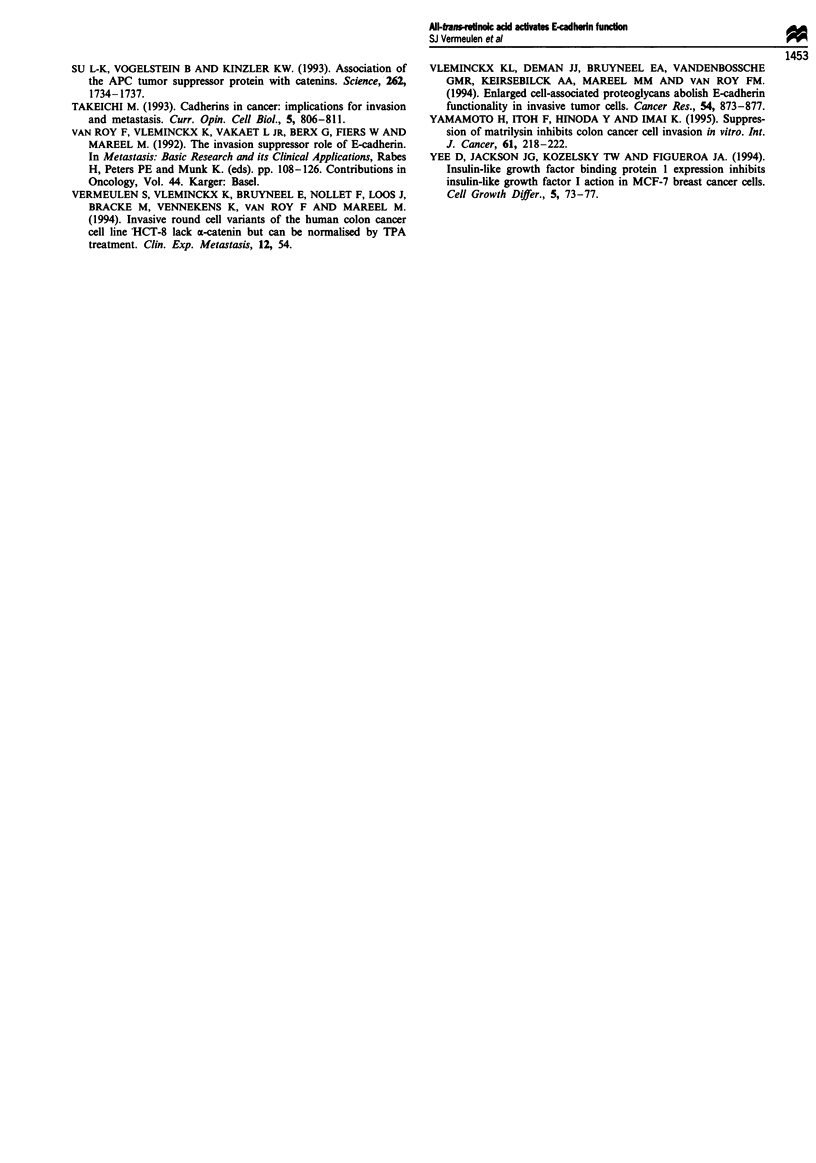# Activation of the E-cadherin/catenin complex in human MCF-7 breast cancer cells by all-trans-retinoic acid.

**DOI:** 10.1038/bjc.1995.528

**Published:** 1995-12

**Authors:** S. J. Vermeulen, E. A. Bruyneel, F. M. van Roy, M. M. Mareel, M. E. Bracke

**Affiliations:** Department of Radiotherapy, Nuclear Medicine and Experimental Cancerology, University Hospital, Gent, Belgium.

## Abstract

**Images:**


British Journal of Cancer (1995) 72, 1447–1453
© 1995 Stockton Press All rights reserved 0007–0920/95 $12.00

### Activation of the E-cadherin/catenin complex in human MCF-7 breast cancer cells by all-*trans*-retinoic acid

SJ Vermeulen[1], EA Bruyneel[1], FM van Roy[2], MM Mareel[1] and ME Bracke[1]

[1]*Department of Radiotherapy, Nuclear Medicine and Experimental Cancerology, University Hospital, De Pintelaan 185, B-9000 Gent, Belgium;* [2]*Laboratory of Molecular Cell Biology, Department of Molecular Genetics, University of Gent, K.L. Ledeganckstraat 35, B-9000 Gent, Belgium.*

**Summary** All-*trans*-retinoic acid (RA), like insulin-like growth factor I (IGF-I) and tamoxifen, inhibit invasion of human MCF-7/6 mammary cancer cells *in vitro*. For tamoxifen and for IGF-I, activation of the invasion-suppressor function of the E-cadherin/catenin complex was shown to be the most probable mechanism of the anti-invasive action. We did a series of experiments to determine whether the anti-invasive effect of RA also implicated the invasion-suppressor E-cadherin/catenin complex. Human MCF-7/6 mammary and HCT-8/R1 colon cancer cells, both with a dysfunctional E-cadherin/catenin complex, were treated with RA and the function of the complex was evaluated through $Ca^{2+}$-dependent fast aggregation. Fast aggregation of both MCF-7/6 and HCT-8/R1 cells was induced by 1 μM RA. This effect was abolished by antibodies against E-cadherin. RA-induced fast aggregation was not sensitive to cycloheximide, tyrosine kinase inhibitors or antibodies against IGF-I or against the IGF-I receptor. RA did not stimulate IGF-I receptor phosphorylation or alter the E-cadherin/catenin complex, as evidenced by immunoprecipitation. RA up-regulates the function of the invasion–suppressor complex E-cadherin/catenin. Its action mechanism is different from that of IGF-I. RA may act as an anti-invasive agent with unique mechanisms of action.

**Keywords:** All-*trans*-retinoic acid; E-cadherin/catenin complex; fast aggregation; invasion

The epithelial $Ca^{2+}$-dependent cell–cell adhesion molecule E-cadherin has an invasion-suppressor function when linked to the actin cytoskeleton via α-catenin plus β-, or γ-catenin (Takeichi, 1993; Mareel *et al.*, 1994). The adhesive function of the E-cadherin/catenin complex can be down-regulated at the transcriptional, translational and post-translational level in experimental (Shimoyama *et al.*, 1992; Behrens *et al.*, 1993) and in human (Bringuier *et al.*, 1993) cancer. Recently, the APC protein has been implicated in the regulation of the E-cadherin/catenin complex through association with α- and β-catenin in the cytoplasm (Su *et al.*, 1993). Furthermore, new models have been described for the homophilic interactions between the extracellular domains of cadherins (Overduin *et al.*, 1995; Shapiro *et al.*, 1995). Experiments both *in vivo* (Mareel *et al.*, 1991) and *in vitro* (Van Roy *et al.*, 1992; Bracke *et al.*, 1993) have suggested that the invasion-suppressor function of the E-cadherin/catenin complex is modulated by external factors. In human MCF-7/6 breast cancer cells IGF-I (Bracke *et al.*, 1993) and the anti-estrogen tamoxifen (Bracke *et al.*, 1994*a*) up-regulated the adhesive function of the E-cadherin/catenin complex and inhibited invasion *in vitro*. Inhibition of invasion of MCF-7/6 cells was also obtained with RA (Bracke *et al.*, 1991). We therefore wanted to examine whether RA could up-regulate the adhesive function of the E-cadherin/catenin complex. We have also examined the effect of RA on the components of the E-cadherin/catenin complex, using immunoprecipitation of metabolically labelled cells. Finally, since the literature mentions that RA modulates IGF-I as well as IGF-binding proteins (IGFBPs) (Fontana *et al.*, 1991; Figueroa and Yee, 1992), we have tested possible relationships between IGF-I-mediated regulation of E-cadherin/catenin functions and effects of RA. The general purpose of our work is to find agents that activate invasion-suppressor molecules and are therefore candidates for chronic anti-invasive treatment of cancer.

Correspondence: MM Mareel
Received 9 March 1995; revised 29 June 1995; accepted 25 July 1995

### Materials and methods

### *Cells*

The MCF-7/6 cell line (obtained from Dr H Rochefort, Unité d'Endocrinologie Cellulaire et Moléculaire, Montpellier, France) is a variant of the human MCF-7 breast cancer cell family. MCF-7/6 cells were treated with 1 μg $ml^{-1}$ mycoplasma removal agent (ICN Biomedicals, Costa Mesa, CA, USA) for seven passages. For the present experiments the cells were harvested from mycoplasma-free stock cultures maintained as described previously (Bracke *et al.*, 1991). The HCT-8/R1 cell line is a subclone from the human HCT-8 colon cancer cell line (CCL244, ATCC, Rockville, MD, USA) that was selected for its round morphotype. HCT-8/R1 cells were maintained in RPMI-1640, supplemented with 1 mM sodium pyruvate and 100 μg $ml^{-1}$ streptomycin. MCF-7/6 (Bracke *et al.*, 1991, 1993) and HCT-8/R1 cells (Vermeulen *et al.*, 1994) are invasive and have a dysfunctional E-cadherin/catenin complex, i.e. unable to mediate fast $Ca^{2+}$-dependent homotypic aggregation. MDA-MB-231 (ATCC; HTB26) cells were maintained in Leibovitz-15 medium, supplemented with 0.05% glutamine. These cells do not express E-cadherin (Frixen *et al.*, 1991). All culture media (Gibco, Gent, Belgium) were supplemented with 10% fetal bovine serum (FBS) and 250 IU $ml^{-1}$ penicillin.

### *Drugs*

All-*trans*-retinoic acid (RA; Sigma, St Louis, MO, USA) was dissolved in ethanol at 1 mM and used at concentrations between 0.1 nM and 1 μM. Control cultures were treated with corresponding ethanol concentrations. To study the role of *de novo* protein synthesis cells were treated with cycloheximide (Sigma) at 10 μg $ml^{-1}$. Recombinant human IGF-I was from Boehringer Mannheim (Brussels, Belgium). As tyrosine kinase inhibitors (all from Gibco) we used Genistein (25 μM), Me-2,5-dihydroxycinnamate (50 μM), RCAM-lysozyme (1 μM) and 2-OH-5-(2,5-diOH-benzyl)aminobenzoic acid (10 μM). Treatment schedules are mentioned in the Results section.

*Antibodies*

MB2 (Bracke *et al.*, 1993) and HECD-1 (Takara Shuzo, Kyoto, Japan) are monoclonal antibodies against human E-cadherin with neutralising effects on E-cadherin functions (Bracke *et al.*, 1993). αIR3 and 82-9A (Oncogene Science, Uniondale, NY, USA), are monoclonal antibodies functionally blocking respectively the human IGF-I receptor (Cullen *et al.*, 1990; Bracke *et al.*, 1993) and human IGF-I (Kerr *et al.*, 1990). PY20 (ICN Biomedicals) is a monoclonal antibody recognising phosphotyrosine. 5D10 (obtained from Dr L Plessers, Limburgs Universitair Centrum, Diepenbeek, Belgium), is a monoclonal antibody against MCF-7 cell surface components (Plessers *et al.*, 1986). Anti CEA (Dakopatts, Glostrup, Denmark), a purified immunoglobulin fraction of rabbit antiserum, reacts with CEA and CEA-like molecules. We prepared rabbit anti-β-catenin antiserum using as an immunogen a synthetic peptide C-PGDSNQLAW-FDTDL (provided by J Vandekerckhove, Laboratory of Physiological Chemistry, University of Gent, Gent, Belgium) corresponding to the C-terminal part of mouse β-catenin (Butz *et al.*, 1992). The peptide was coupled on keyhole limpet haemocyanin via the additional N-terminal cysteine and used for four cycles of intradermal and intramuscular immunisation of rabbits. Immune serum was affinity purified by fast protein liquid chromatography (FPLC) (Pharmacia, Uppsala, Sweden) with the peptide bound to a *p*-hydroxymercuribenzoate matrix (Sigma). Immune serum 1522 recognised a 95 kDa band on Western blots and immunoprecipitated from metabolically labelled cell lysates the 95, 102 and 120 kDa bands that are specific for the E-cadherin/catenin complex.

*Assay for fast aggregation*

Cell–cell adhesion was numerically evaluated in an aggregation assay as described (Bracke *et al.*, 1993). Briefly, cells were detached in E-cadherin-saving conditions and allowed to aggregate on a Gyrotory shaker (New Brunswick Scientific, New Brunswick, NJ, USA) in a buffer containing 1.25 mM $Ca^{2+}$ and 0.1% bovine serum albumin. The aggregation index was expressed as $1 - N_{30}/N_0$, where $N_0$ indicates the initial number of particles and $N_{30}$ the number of particles after 30 min present in a constant volume of 500 μl. The number of particles was measured by a Coulter counter ZM (Coulter, Miami, FL, USA) with the following settings: full scale, 10 mA; current, 3.34 mA; lower threshold, 5.0 μM; attenuation, 32; preset gain, 2; aperture size, 140 μm. Aggregate size distribution in function of volume % (total volume of all aggregates equals 100%) was analysed on 20 000 particles by a Coulter MultisizerIIe with the following settings: current, 3.2 mA; lower threshold, 8.0 μM; preset gain, 1; aperture size, 400 μm.

*Flow cytometric analysis of E-cadherin*

MCF-7/6 cells, detached as in the assay for fast aggregation, were incubated with the antibody against E-cadherin (HECD-1) followed by rabbit anti-mouse antiserum conjugated with fluoresceine isothiocyanate (RAM-FITC, Dakopatts) as a second antibody (Bracke *et al.*, 1993). Fluorescence intensity was measured with a FACScan III (Becton Dickinson, Mountain View, CA, USA).

*Molecular characterisation of the E-cadherin/catenin complex*

The E-cadherin/catenin complex was characterised by co-immunoprecipitation from metabolically labelled cells as described (Vleminckx *et al.*, 1994) with the following modifications. Cells on plastic tissue culture substrate were washed three times in methionine-free Eagle's minimum essential medium (EMEM, Gibco) with 2% dialysed FBS (Gibco) followed by incubation for 30 min in the same medium and addition of 100 μCi $ml^{-1}$Tran$^{35}$S (ICN Biomedicals) for 3 h. Cells were detached as in the assay for fast aggregation and lysed after 10 min using the lysis buffer described previously with the following protease inhibitors (all from Sigma): phenylmethylsulphonyl fluoride (1.72 mM), leupeptin (21 μM), aprotinin (10 μg $ml^{-1}$). Equal amounts of trichloroacetic acid-precipitable material were precleared for 30 min at 4°C with 25 μl protein G sepharose 4 fast flow (Pharmacia). HECD-1 (1 μg per precipitation) or immune serum 1522 (20 μl per precipitation) followed by protein G sepharose 4 fast flow were used for co-immunoprecipitation. The immune complexes were washed three times with 750 μl of lysis buffer. Proteins were eluted by sodium dodecyl sulphate under reducing conditions and analysed by sodium dodecyl sulphate polyacrylamide (6%) gel electrophoresis followed by fluorography.

*Molecular characterisation of IGF-I receptor and binding proteins*

The IGF-I receptor phosphorylation was evaluated by sequential immunoprecipitation as described by Izumi *et al.* (1987) with the following modifications. Cells were washed three times in phosphate-free EMEM with 2% dialysed FBS (Gibco) followed by labelling with 0.5 mCi $ml^{-1}$ carrier-free [$^{32}$P]orthophosphate (Amersham, Gent, Belgium) for 2 h. Cells were lysed in the presence of the following phosphatase inhibitors (all from Sigma): sodium pyrophosphate (10 mM); sodium fluoride (10 mM); sodium vanadate (1 mM). Phosphotyrosine molecules were immunoprecipitated with PY20 (5 μg per precipitation), immune complexes were washed three times with 750 μl of lysis buffer and molecules were eluted three times for 15 min with 250 μl elution buffer containing: phospho-L-tyrosine (10 mM) and phenylphosphate (10 mM) in lysis buffer. Eluted molecules were immunoprecipitated with αIR3 (1 μg per precipitation) and proteins were analysed as described with the E-cadherin/catenin complex. IGFBPs were evaluated by ligand blotting (Fontana *et al.*, 1991). Briefly, cells were washed 5 times with serum-free medium followed by incubation for 44 h in the same medium. The conditioned medium was dialysed against 1.5 mM Tris and concentrated 100 times by lyophilisation. Lyophilised proteins were denatured with sodium dodecyl sulphate under non-reducing conditions, separated on 12% polyacrylamide gels and electroblotted. IGFBPs were visualised by [$^{125}$I]IGF-I (Hossenlopp *et al.*, 1986). We quantitated IGFBPs with an XRS 12cx Omnimedia scanner using Bio Image Whole Band Analyser software (Millipore, Etten-Leur, The Netherlands) on a Sun Sparc Classic computer.

**Figure 1** Fast aggregation ($1 - N_{30}/N_0$) of MCF-7/6, HCT-8/R1 and MDA-MB-231 cells with ( + ) or without ( – ) $Ca^{2+}$. Cells were treated with RA at 1 μM ( + ) or with solvent ( – ) for 4 ($^a$) or 24 h ($^b$) and the agent was present during aggregation. Cells were pretreated for 30 min at 4°C with( + ) or without ( – ) the E-cadherin-specific antibody MB2 or HECD-1 (diluted 1:20) and the antibodies remained present during aggregation. Bars represent mean values + s.d. of six measurements.

### Results

MCF-7/6 cells showed a poor tendency to aggregate in accordance with previous results (Bracke *et al.*, 1993). Pretreatment of cells with RA at 1 μM induced $Ca^{2+}$-dependent and E-cadherin-specific fast aggregation of MCF-7/6 cells (Figure 1). Analysis of aggregate size distribution showed formation of larger aggregates in RA-treated than in untreated samples (Figure 2). The effect of RA was counteracted by an antibody against E-cadherin (Figure 2). The minimum duration of treatment needed for maximum effect was 4 h. Partial response was obtained within 2 h of treatment at a concentration of 1 μM RA or within 24 h treatment at 0.1 μM (Figure 3). RA also induced E-cadherin-specific fast aggregation in HCT-8/R1 cells lacking α-catenin, although to a lesser extent than in MCF-7/6 cells (Figure 1). The E-cadherin-negative MDA-MB-231 cells showed $Ca^{2+}$-independent fast aggregation that was hardly altered by RA or by an antibody against E-cadherin (Figure 1). RA-induced fast aggregation of MCF-7/6 cells was not inhibited by cycloheximide at concentrations that reduced Tran$^{35}$S incorporation to less than 15% of untreated controls (Figure 4). It was lowered by antibodies functionally blocking E-cadherin but not by antibodies against other surface molecules, (Figure 5) shown to be expressed on MCF-7/6 cells by Western blots (CEA, 5D10; data not shown). Neither did RA-induced fast aggregation respond to antibodies against IGF-I or IGF-I receptor (Figure 5) in matched experiments in which IGF-I-induced aggregation was clearly inhibited (data not shown). RA changed neither the level of E-cadherin expressed at the cell surface as revealed by flow cytometry (Figure 6) nor the composition of the E-cadherin/catenin complex in MCF-7/6 cells (Figure 7). IGF-I receptor phosphorylation was not increased by RA in contrast to the effect of IGF-I (Figure 8). Neither was RA-induced fast aggregation inhibited by the

**Figure 2** Aggregate size distribution of RA-induced fast aggregation of MCF-7/6 cells. Approximately 20 000 particles were analysed before (□) and after 30 min (◆) of aggregation. Cells were treated (**b** and **c**) or not (**a**) with 1 μM RA for 24.5 h, including the time of aggregation. Cells were pretreated (**c**) for 1 h at 4°C with the E-cadherin-specific antibody MB2 (diluted 1:20) and the antibodies remained present during aggregation. Ordinate, percentage of total volume of aggregates; abscissa, size of aggregates in μm.

**Figure 3** RA-induced fast aggregation ($1 - N_{30}/N_0$) of MCF-7/6 cells as a function of RA concentration (**a**) and time of RA treatment (**b**). (**a**) Treatment with RA (concentration in μM) for 24.5 h including the time of aggregation. (**b**) Treatment with 1 μM RA (time in h). Bars represent mean values + s.d. of six measurements.

**Figure 4** Fast aggregation ($1 - N_{30}/N_0$; open bars) and Tran$^{35}$S incorporation (percentage of untreated; hatched bars) by MCF-7/6 cells pretreated ( + ) or not ( – ) with 10 μg ml$^{-1}$ cycloheximide for 5 h and with 1 μM RA ( + ) or with solvent ( – ) during the last 4 h; when cycloheximide and RA were added, they were also present during the aggregation assay. Bars represent mean values + s.d. of six measurements.

**Figure 5** RA-induced fast aggregation ($1 - N_{30}/N_0$) of MCF-7/6 cells in the presence of antibodies against E-cadherin (HECD-1, 1:20), CEA (anti-CEA, 1:100), an unidentified MCF-7 cell surface epitope (5D10, 1:10), the IGF-I receptor (αIR3; 15 μg ml$^{-1}$) or IGF-I (82-9A, 15 μg ml$^{-1}$). Treatment with RA (1 μM) was for 4.5 h including the time of aggregation. Cells were pretreated with antibodies for 5 h (82-9A or αIR3) at 37°C or for 30 min (all other antibodies) at 4°C and antibodies remained present during aggregation. Bars represent mean values + s.d. of six measurements.

tyrosine kinase inhibitors Genistein (25 μM), Me-2,5-dihydroxycinnamate (50 μM), RCAM-lysozyme (1 μM) or 2-OH-5-(2,5-diOH-benzyl)aminobenzoic acid (10 μM) (Table I), although such concentrations inhibited IGF-I-induced fast aggregation (Bracke *et al.*, 1994*b*). The total amount of IGFBPs in medium conditioned for 44 h from MCF-7/6 cells treated with RA at 0.1 μM was 1.1 and 1.5 times (two independent experiments) higher than in medium from untreated cultures.

**Figure 6** Flow cytometric analysis of E-cadherin expression in MCF-7/6 cells treated (**b**) or not (**a**) with 1 μM RA for 4 h. The anti-E-cadherin antibody HECD-1 (1:20) was used (open area) or not (shaded area) as first antibody; RAM-FITC was used (1:20) as second antibody. Ordinate, number of cells; abscissa, fluorescence intensity.

### Discussion

We report that RA, at anti-invasive concentrations, induces fast aggregation of cells which have a dysfunctional E-cadherin/catenin complex. This induction was prevented by monoclonal antibodies that functionally blocked E-cadherin but not by other antibodies also binding to the cell surface. For MCF-7/6 breast cancer cells, induction of E-cadherin-dependent fast aggregation was achieved also with IGF-I (Bracke *et al.*, 1993), with tamoxifen (Bracke *et al.*, 1994*a*) and with the citrus flavonoid tangeretin (Bracke *et al.*, 1994*b*). All these agents inhibited invasion of MCF-7/6 cells in organ culture confirming the invasion-suppressor function of the E-cadherin/catenin complex (Mareel *et al.*, 1994). E-cadherin is known to act as an organiser of junctional complexes. It might be that the rapid and reversible cell type- and concentration-related up- or down-modulation of gap–junctional communication by RA (Mehta *et al.*, 1989) occurs via modulation of the E-cadherin/catenin complex. Induction of the epithelioid morphotype and of E-cadherin expression at the cell–cell contact sites was reported with human SK-BR-3 mammary cancer cells treated with RA (Anzano *et al.*, 1994).

RA-induced fast aggregation also occurred in the absence of *de novo* protein synthesis, as demonstrated by our experiments with cycloheximide. This suggests that RA, in association with its receptors or not, interacts directly with the E-cadherin/catenin complex or its effectors. It does not, however, exclude the possibility that RA acts via binding to hormone-sensitive elements, leading to arrest of transcription of an inhibitory protein. The fact that cycloheximide by itself has no effect on fast aggregation argues against the latter possibility.

The relatively high concentrations (1–0.1 μM) of RA needed to induce fast aggregation are similar to those described for the decrease in B16 melanoma cell aggregation (Edward *et al.*, 1992) and for down-regulation of β4 integrins in LL4 cells (Gaetano *et al.*, 1994). This need may be ascribed to the presence of serum in the culture medium and of albumin in the salt solution used for the aggregation assay (see Materials and methods). Albumin is known to bind RA as it serves as its carrier protein in the blood (Allen and Bloxham, 1989).

Our co-immunoprecipitation data failed to demonstrate an effect of RA on the composition of the E-cadherin/catenin complex. This observation suggests that the cause of dysfunction of E-cadherin in MCF-7/6 cells is situated downstream of the E-cadherin/catenin complex. The fact that RA also induced E-cadherin-dependent fast aggregation, although less effectively, in α-catenin-deficient HCT-8/R1 cells supports the idea of a downstream defect in MCF-7/6 cells.

RA does not seem to interact directly with IGFBPs, IGF-I or the IGF-I receptor, all of which are implicated in IGF-I-mediated aggregation of MCF-7/6 cells (Bracke *et al.*, 1993). An action via an autocrine IGF-I loop is unlikely because antibodies functionally blocking IGF-I did not hamper RA-induced aggregation. Neither could it be inhibited with antibodies against the IGF-I receptor nor did phosphorylation of the IGF-I receptor occur upon RA treatment in contrast to addition of IGF-I. Moreover, the tyrosine kinase inhibitors that blocked IGF-I-induced fast aggregation had no effect on RA-induced aggregation. It is unlikely that the slight increase in IGFBPs found in our and in others' experiments (Fontana *et al.*, 1991; Yee *et al.*, 1994) was involved in RA-induced aggregation, since such an increase would have had an opposite effect. Indeed, IGFBPs are known to neutralise IGF-I and a variant of IGF-I lacking the IGFBP-binding domain was much more potent than genuine IGF-I at inducing fast aggregation (Bracke *et al.*, 1994*b*).

Our present results indicate that activation of the E-cadherin/catenin complex may contribute to the anti-invasive

**Figure 7** Autoradiographs of sodium dodecyl sulphate polyacrylamide gel electropherograms from total lysates of MCF-7/6 cells metabolically labelled with Tran$^{35}$S and immunoprecipitated with the E-cadherin-specific antibody HECD-1 (top), with the rabbit anti-β-catenin antiserum 1522 (middle) or without antibody (bottom). MCF-7/6 cells were treated for 2 h with 1 μM RA (lane 1), with solvent (lane 2) or untreated (lane 3). E-CAD, E-cadherin; α-CTN, α-catenin; β-CTN, β-catenin; γ-CTN, γ-catenin. Horizontal bars (right) indicate molecular weight markers.

**Figure 8** Autoradiograph of sodium dodecyl sulphate polyacrylamide gel electropherogram from total lysates of MCF-7/6 cells metabolically labelled with $^{32}$P and sequentially immunoprecipitated with respectively, a phosphotyrosine specific antibody (PY20) and an IGF-I receptor specific antibody (αIR3). MCF-7/6 cells were treated for 4 h with 1 μM RA (lane 1), with 250 μM acetic acid (solvent of IGF-1, lane 2) or 0.5 μg ml$^{-1}$ IGF-I (lane 3). β, β-Subunit of the IGF-I receptor. Horizontal bars (right) indicate molecular weight markers.

**Table I** Fast aggregation of MCF-7/6 cells

| *Treatment*[a] | $1 - N_{30}/N_0$[b] *RA*[c] | *IGF-I*[d] |
|---|---|---|
| None | 0.469 ± 0.004 | 0.274 ± 0.005 |
| 10 μM 2-OH-5-[2,5-diOH-benzyl] aminobenzoic acid | 0.487 ± 0.014 | 0.015 ± 0.003 |
| 1 μM RCAM-lysozyme | 0.456 ± 0.019 | 0.038 ± 0.008 |
| 50 μM Me-2,5-diOH-cinnamate | 0.613 ± 0.020 | 0.041 ± 0.013 |
| 25 μM Genistein | 0.633 ± 0.007 | 0.033 ± 0.006 |

([a]) Cells were treated with tyrosine kinase inhibitors 1 h preceding and during RA or IGF-1 treatment. ([b]) Aggregation index, numerical values indicate mean ± s.d. of six measurements. Cells were treated with ([c]) 1 μM RA for 4.5 h or ([d]) 0.5 μg ml$^{-1}$ IGF-I for 30 min. In the absence of either tyrosine kinase inhibitor, RA or IGF-I, MCF-7/6 aggregation was: 0.080 ± 0.008 (mean ± s.d. of six measurements).

activity of RA on MCF-7/6 cells as described previously (Bracke *et al*., 1991). It is, however, unlikely that this is the only mechanism of the anti-invasive action of RA. Such action was described also for melanoma (Helige *et al*., 1993) and rhabdomyosarcoma (Gerharz *et al*., 1993) cells which are not expected to express E-cadherin. RA did not inhibit, however, the invasion of the E-cadherin-negative MDA-MB-231 cells into chick heart (our unpublished results). This shows the existence of alternative mechanisms of anti-invasiveness such as inhibition of proteolytic enzymes (Gudas *et al*., 1994; Yamamoto *et al*., 1995).

Taken together, our results indicate that RA-induced aggregation of MCF-7/6 cells via the E-cadherin/catenin complex depends upon a mechanism other than IGF-I-induced aggregation. This novel function, namely the activation of a dysfunctional E-cadherin/catenin complex via a protein synthesis-independent mechanism, might identify RA as a potential anti-invasive agent for combinatorial cancer treatment.

**Acknowledgements**
We thank M Goethals for help in preparation of antibody, L Baeke, R Colman, K Vennekens and A Verspeelt for technical assistance, J Roels for preparation of the illustrations and W Dewispelaere (N.V. Analis, Gent, Belgium) for the measurements on the Coulter MultisizerIIe. This work was supported by the State of Florida Department of Citrus, the Nationaal Fonds voor Wetenschappelijk Onderzoek, the Vereniging voor Kankerbestrijding VZW, the Sportvereniging tegen Kanker, the Effel Schenking, the ASLK/VIVA-verzekeringen, the GOA van de Vlaamse Gemeenschap, Brussels, Belgium.

**References**

ALLEN JG AND BLOXHAM DP. (1989). The pharmacology and pharmacokinetics of the retinoids. *Pharmacol. Ther*., **40**, 1–27.

ANZANO MA, BYERS SW, SMITH JM, PEER CW, MULLEN LT, BROWN CC, ROBERTS AB AND SPORN MB. (1994). Prevention of breast cancer in the rat with 9-*cis*-retinoic acid as a single agent and in combination with tamoxifen. *Cancer Res*., **54**, 4614–4617.

BEHRENS J, VAKAET L, FRIIS R, WINTERHAGER E, VAN ROY F, MAREEL MM AND BIRCHMEIER W. (1993). Loss of epithelial morphotype and gain of invasiveness correlates with tyrosine phosphorylation of the E-cadherin/β-catenin complex in cells transformed with a temperature-sensitive v-*src* gene. *J. Cell Biol*., **120**, 757–766.

BRACKE ME, VAN LAREBEKE NA, VYNCKE BM AND MAREEL MM. (1991). Retinoic acid modulates both invasion and plasma membrane ruffling of MCF-7 human mammary carcinoma cells *in vitro*. *Br. J. Cancer*, **63**, 867–872.

BRACKE ME, VYNCKE BM, BRUYNEEL EA, VERMEULEN SJ, DE BRUYNE GK, VAN LAREBEKE NA, VLEMINCKX K, VAN ROY FM AND MAREEL MM. (1993). Insulin-like growth factor I activates the invasion suppressor function of E-cadherin in MCF-7 human mammary carcinoma cells *in vitro*. *Br. J. Cancer*, **68**, 282–289.

BRACKE ME, CHARLIER C, BRUYNEEL EA, LABIT C, MAREEL MM AND CASTRONOVO V. (1994*a*). Tamoxifen restores the E-cadherin function in human breast cancer MCF-7/6 cells and suppresses their invasive phenotype. *Cancer Res*., **54**, 4607–4609.

BRACKE ME, VERMEULEN SJ, BRUYNEEL EA, VENNEKENS KM, DE BRUYNE GK, VAN ROY FM AND MAREEL MM. (1994*b*). The invasion suppressor function of E-cadherin in mammary epitheloid cells. In *Prospects in Diagnosis and Treatment of Breast Cancer*, Schmitt M, Graeff H and Kindermann G. (eds) pp. 107–115. Exerpta Medica International Congress Series No. 1050, Elsevier: Amsterdam.

BRINGUIER PP, UMBAS R, SCHAAFSMA HE, KARTHAUS HFM, DEBRUYNE FMJ AND SCHALKEN JA. (1993). Decreased E-cadherin immunoreactivity correlates with poor survival in patients with bladder tumors. *Cancer Res*., **53**, 3241–3245.

BUTZ S, STAPPERT J, WEISSIG H AND KEMLER R. (1992). Plakoglobin and β-catenin: distinct but closely related. *Science*, **257**, 1142–1144.

CULLEN KJ, YEE D, SLY WS, PERDUE J, HAMPTON B, LIPPMAN ME AND ROSEN N. (1990). Insulin-like growth factor receptor expression and function in human breast cancer. *Cancer Res*., **50**, 48–53.

EDWARD M, GOLD JA AND MACKIE RM. (1992). Retinoic acid-induced inhibition of metastatic melanoma cell lung colonization and adhesion to endothelium and subendothelial extracellular matrix. *Clin. Exp. Metastasis*, **10**, 61–67.

FIGUEROA JA AND YEE D. (1992). The insulin-like growth factor binding proteins (IGFBPs) in human breast cancer. *Breast Cancer Res. Treat*., **22**, 81–90.

FONTANA JA, BURROWS-MEZU A, CLEMMONS DR AND LEROITH D. (1991). Retinoid modulation of insulin-like growth factor-binding proteins and inhibition of breast carcinoma proliferation. *Endocrinology*, **128**, 1115–1122.

FRIXEN UH, BEHRENS J, SACHS M, EBERLE G, VOSS B, WARDA A, LÖCHNER D AND BIRCHMEIER W. (1991). E-cadherin-mediated cell–cell adhesion prevents invasiveness of human carcinoma cells. *J. Cell Biol*., **113**, 173–185.

GAETANO C, MELCHIORI A, ALBINI A, BENELLI R, FALCIONI R, MODESTI A, MODICA A, SCARPA S AND SACCHI A. (1994). Retinoic acid negatively regulates β4 integrin expression and suppresses the malignant phenotype in a Lewis lung carcinoma cell line. *Clin. Exp. Metastasis*, **12**, 63–72.

GERHARZ CD, BRACKE ME, MAREEL MM AND GABBERT HE. (1993). Modulation of invasive potential in different clonal subpopulations of a rat rhabdomyosarcoma cell line (BA-HAN-1) by differentiation induction. *Clin. Exp. Metastasis*, **11**, 55–67.

GUDAS LJ, SPORN MB AND ROBERTS AB. (1994). Cellular biology and biochemistry of the retinoids. In *The Retinoids: Biology, Chemistry and Medicine*, 2nd edn., Sporn MB, Roberts AB and Goodman DS. (eds), pp. 443–520. Raven Press Ltd: New York.

HELIGE C, SMOLLE J, ZELLNIG G, HARTMANN E, FINK-PUCHES R, KERL H AND TRITTHART HA. (1993). Inhibition of K1735-M2 melanoma cell invasion *in vitro* by retinoic acid. *Clin. Exp. Metastasis*, **11**, 409–418.

HOSSENLOPP P, SEURIN D, SEGOVIA-QUINSON B, HARDOUIN S AND BINOUX M. (1986). Analysis of serum insulin-like growth factor binding proteins using Western blotting: use of the method for titration of the binding proteins and competitive binding studies. *Anal. Biochem*., **154**, 138–143.

IZUMI T, WHITE MF, KADOWAKI T, TAKAKU F, AKANUMA Y AND KASUGA M. (1987). Insulin-like growth factor I rapidly stimulates tyrosine phosphorylation of a $M_r$ 185,000 protein in intact cells. *J. Biol. Chem*., **262**, 1282–1287.

KERR DE, LAARVELD B AND MANNS JG. (1990). Effects of passive immunization of growing guinea-pigs with an insulin-like growth factor-I monoclonal antibody. *J. Endocrinol*., **124**, 403–415.

MAREEL MM, BEHRENS J, BIRCHMEIER W, DE BRUYNE GK, VLEMINCKX K, HOOGEWIJS A, FIERS WC AND VAN ROY FM. (1991). Downregulation of E-cadherin expression in Madin Darby canine kidney (MDCK) cells inside nude mice tumors. *Int. J. Cancer*, **47**, 922–928.

MAREEL M, BRACKE M AND VAN ROY F. (1994). Invasion promoter versus invasion suppressor molecules: the paradigm of E-cadherin. *Mol. Biol. Rep*., **19**, 45–67.

MEHTA PP, BERTRAM JS AND LOEWENSTEIN WR. (1989). The actions of retinoids on cellular growth correlate with their actions on gap junctional communication. *J. Cell Biol*., **108**, 1053–1065.

OVERDUIN M, HARVEY TS, BAGBY S, TONG KI, YAU P, TAKEICHI M AND IKURA M. (1995). Solution structure of the epithelial cadherin domain responsible for selective cell adhesion. *Science*, **267**, 386–389.

PLESSERS L, BOSMANS E, COX A AND RAUS J. (1986). Specific monoclonal antibodies reacting with human breast cancer cells. *Anticancer Res*., **6**, 885–888.

SHAPIRO L, FANNON AM, KWONG PD, THOMPSON A, LEHMANN MS, GRÜBEL G, LEGRAND JF, ALS-NIELSEN J, COLMAN DR AND HENDRICKSON WA. (1995). Structural basis of cell–cell adhesion by cadherins. *Nature*, **374**, 327–337.

SHIMOYAMA Y, NAGAFUCHI A, FUJITA S, GOTOH M, TAKEICHI M, TSUKITA S AND HIROHASHI S. (1992). Cadherin dysfunction in a human cancer cell line: possible involvement of loss of α-catenin expression in reduced cell–cell adhesiveness. *Cancer Res*., **52**, 5770–5774.

SU L-K, VOGELSTEIN B AND KINZLER KW. (1993). Association of the APC tumor suppressor protein with catenins. *Science*, **262**, 1734–1737.

TAKEICHI M. (1993). Cadherins in cancer: implications for invasion and metastasis. *Curr. Opin. Cell Biol.*, **5**, 806–811.

VAN ROY F, VLEMINCKX K, VAKAET L JR, BERX G, FIERS W AND MAREEL M. (1992). The invasion suppressor role of E-cadherin. In *Metastasis: Basic Research and its Clinical Applications*, Rabes H, Peters PE and Munk K. (eds). pp. 108–126. Contributions in Oncology, Vol. 44. Karger: Basel.

VERMEULEN S, VLEMINCKX K, BRUYNEEL E, NOLLET F, LOOS J, BRACKE M, VENNEKENS K, VAN ROY F AND MAREEL M. (1994). Invasive round cell variants of the human colon cancer cell line HCT-8 lack α-catenin but can be normalised by TPA treatment. *Clin. Exp. Metastasis*, **12**, 54.

VLEMINCKX KL, DEMAN JJ, BRUYNEEL EA, VANDENBOSSCHE GMR, KEIRSEBILCK AA, MAREEL MM AND VAN ROY FM. (1994). Enlarged cell-associated proteoglycans abolish E-cadherin functionality in invasive tumor cells. *Cancer Res.*, **54**, 873–877.

YAMAMOTO H, ITOH F, HINODA Y AND IMAI K. (1995). Suppression of matrilysin inhibits colon cancer cell invasion *in vitro*. *Int. J. Cancer*, **61**, 218–222.

YEE D, JACKSON JG, KOZELSKY TW AND FIGUEROA JA. (1994). Insulin-like growth factor binding protein 1 expression inhibits insulin-like growth factor I action in MCF-7 breast cancer cells. *Cell Growth Differ.*, **5**, 73–77.